# Understanding the factors affecting teachers’ burnout during the COVID-19 pandemic: A cross-sectional study

**DOI:** 10.1371/journal.pone.0279383

**Published:** 2022-12-30

**Authors:** Orly Shimony, Yael Malin, Haya Fogel-Grinvald, Thomas P. Gumpel, Mor Nahum

**Affiliations:** 1 School of Occupational Therapy, Faculty of Medicine, The Hebrew University, Jerusalem, Israel; 2 Seymour Fox School of Education, The Hebrew University, Jerusalem, Israel; University of Catania: Universita degli Studi di Catania, ITALY

## Abstract

**Background:**

During the COVID-19 pandemic, which enforced social distancing and isolation, teachers were required to handle multiple challenges related to their work, including dealing with remote teaching, in addition to personal, medical and financial challenges. The goal of the current research was to examine factors that contributed to professional burnout and commitment to work among teachers during the first and second waves of the COVID-19 pandemic.

**Methods:**

A total of 344 elementary school teachers in Israel completed online self-report questionnaires, including assessments of stressors, anxiety, resilience, self-efficacy beliefs, and coping strategies. Structured Equation Modeling [SEM] was used to examine the contribution of these factors to professional burnout and commitment.

**Results:**

The gaps between needed and received support had a direct effect on teachers’ burnout and commitment, and an indirect effect through anxiety and self-efficacy beliefs. Stress relating to remote teaching and support-gaps regarding remote teaching were the most significant of all the stressors and sources of support.

**Conclusions:**

Collectively, these findings highlight the significance of remote teaching as the main cause of stress and professional burnout and suggest that proper preparation of teachers—before and during times of crisis, may have a significant impact on their mental and professional well-being.

## Introduction

Professional “burnout” has been defined as a prolonged response to chronic emotional and interpersonal stressors on the job [1]. Several studies confirmed a two-factor structure of the burnout syndrome, including emotional exhaustion and personal fulfillment [2, 3]. Teaching is considered a profession with high rates of burnout [4, 5], which eventually lead to high professional turnover rates [6]. Teachers burnout has a significant impact not only on their own will to maintain their profession and their ability to manage classroom behaviors but was also shown to affect their students’ performance and motivation [7]. In this study, we aimed to better understand the factors that contribute to teachers’ burnout and commitment to work during the COVID-19 pandemic.

The COVID-19 pandemic, which was announced in March 2020 by the World Health Organization (WHO) as a worldwide pandemic, was a significant global stressor [8, 9]. As part of the curve-flattening policy adopted by many countries around the globe, schools were closed, affecting more than 1.5 billion students from 185 different countries [10]. As a result, educational systems were forced to adopt emergency routines and new teaching methods, such as remote teaching, or learning with parents’ assistance and involvement [11]. In addition, school teams dealt with health and financial uncertainties and frequent changes in teaching methods. Indeed, several studies to date demonstrated the effect of COVID-19 on teachers’ mental and professional state [9–13]. Collectively, these studies report increases in teacher burnout, which also resulted in high rates of turnover during the pandemic. However, the specific factors within this uniquely stressful situation that may lead to increased burnout rates are still not entirely understood.

Burnout has been shown to be affected by both internal (e.g., psychological distress, anxiety) [14] as well as external factors, such as teaching resources [15]. Considering internal factors, such as distress, and anxiety first, these factors were shown to collectively increase in the general population [16–18] and specifically in teacher populations [13] during the pandemic. Not surprisingly, increased levels of professional burnout were observed among teachers during the pandemic. For example, a study conducted among middle school teachers in Israel found that the high levels of stress during the pandemic were associated with increased burnout and desire to leave the profession [14]. Similarly, a cross-sectional study among healthcare workers found that trait worry and psychological distress significantly predicted work burnout during the pandemic [19]. Another cross-sectional study conducted during the first wave of the pandemic among 125 primary school teachers found that 54% of them experienced burnout [12].

Self-efficacy beliefs, defined as “people’s beliefs about their capabilities to produce designated levels of performance that exercise influence over events that affect their lives” [20], have also been suggested as another internal predictor of teachers’ burnout [21]. Teachers’ self-efficacy beliefs are related to their ability to be effective teachers [22] and to their commitment to teaching [23] and are negatively correlated with emotional exhaustion [24]. The more teachers perceive themselves as empowered by their organization, the more they express their commitment to their organization and to their profession [25]. Specifically, self-efficacy beliefs may act as a mediator of the relationship between the stress experienced by teachers during this period and eventual burnout. Studies found that teachers’ self-efficacy beliefs ratings were lower during the pandemic compared to previous studies conducted before the pandemic [e.g., 26]. Furthermore, teachers who engaged in virtual teaching only, had the lowest levels of self-efficacy beliefs compared to their peers, who taught in hybrid or face-to-face models. This may be related to the challenges of using novel teaching methods or to the stress and anxiety from teaching the pandemic. In contrast, higher self-efficacy beliefs were found among teachers who reported greater levels of support within their schools during the pandemic [26]. However, none of these studies, to the best of our knowledge, has examined the effect of teachers’ self-efficacy beliefs on burnout levels during the pandemic.

The type of specific coping strategy used to deal with the stressful situation is another internal factor that may contribute to teachers’ professional burnout [27]. In distressing situations, people use one of two coping strategies—approach-coping or avoidant-coping. Approach-coping strategies are activities designed to change stressful situations or accept their presence, such as seeking comfort and understanding. In contrast, avoidant-coping strategies aim to increase emotional or physical distancing from stressful situations, such as drug and alcohol use [28]. In a study which examined the coping strategies used by teachers during the COVID-19 lockdowns, the authors found that the approach-coping strategies were linked to increased happiness, welfare, health, and resilience [29]. In contrast, avoidant-coping strategies were associated with higher levels of stress, anxiety, anger, sadness, and loneliness. Herein, we ask whether the specific coping strategy used is associated with teachers’ burnout.

In addition to these internal factors—of anxiety, distress, self-efficacy beliefs, and coping strategies—there are external factors that may also significantly affect teacher burnout. Among them, the social support system within the school seems to be a key factor. Studies show that within-school support from peers and supervisors is more effective in reducing teacher burnout compared with non-school support from family and friends [e.g., 30]. A recent study conducted during the COVID-19 pandemic found that administrative support, such as instructional, technological, or emotional assistance, played a crucial role in reducing teacher burnout [31]. Similarly, perceived support, such as support from the school principal and peer assistance, may also contribute to reducing emotional exhaustion and improving personal accomplishment among teachers [32]. In addition, social support may strengthen the sense of self-efficacy beliefs among teachers, leading to further reduction in burnout levels [33]. A comprehensive study from Canada which included 1,626 teachers found that the changes in teaching methods and administrative support predicted teacher burnout during the pandemic [34]. This is in line with a recent report which examined the educational policy and effects in OECD countries and found that shifting from frontal teaching to remote teaching during the pandemic was done without proper training and support [10]. Collectively, these studies show that increased burnout during the pandemic was the result of new information and communication technologies, and that the support from the school played a key role in the ability to handle these challenges [12].

Teachers’ seniority may also affect their professional burnout, commitment to work, and self-efficacy beliefs. However, findings related to seniority are thus far mixed. For example, while one study found that seniority affected self-efficacy beliefs [33], another study involving elementary school teachers did not find an effect of seniority on burnout [32]. One potential reason for this discrepancy could be that the effect of seniority on burnout is non-linear. Indeed, a study among 201 high-school teachers, found that teachers with up to five years of seniority and teachers with 21 years or more were more committed to their organization compared with teachers with 6–20 years of seniority [33]. To the best of our knowledge, no study to date examined the effect of seniority on teachers’ burnout during the pandemic.

**Fig 1:**
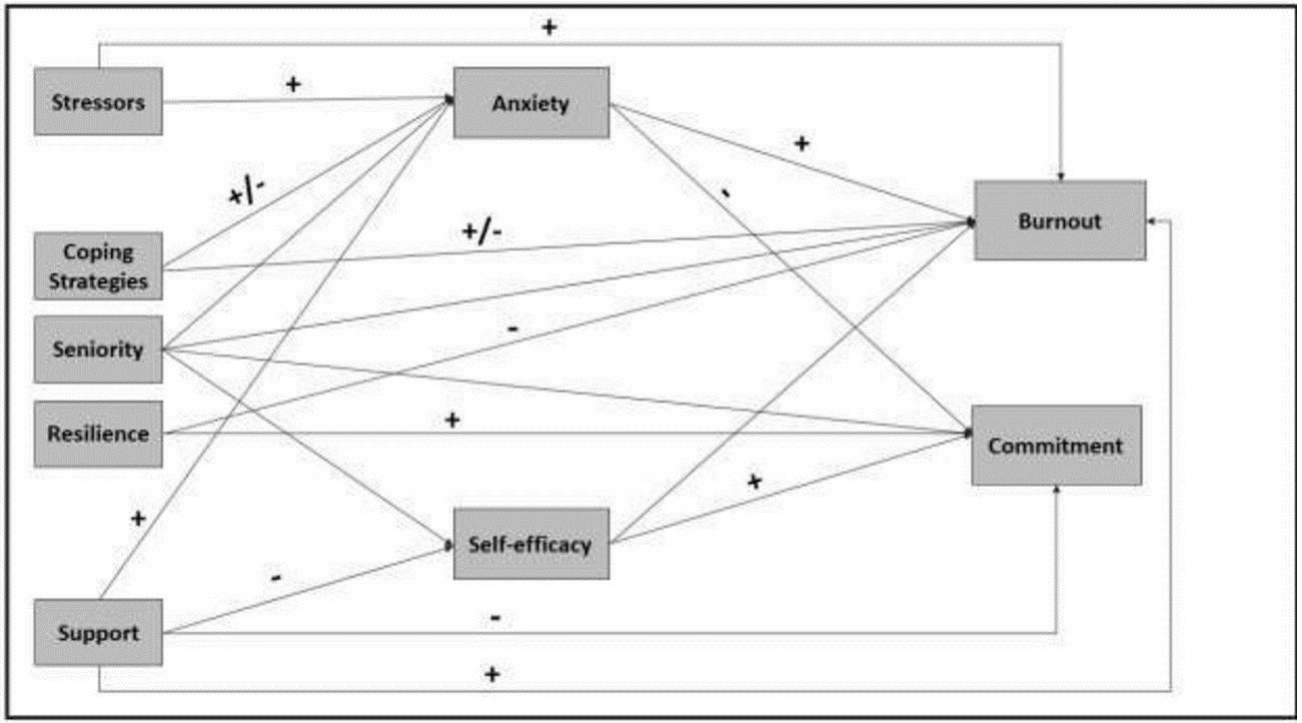
A suggested analytical model showing the hypothesized relationship between factors affecting teachers’ burnout and professional commitment during the pandemic. Hypothesized positive correlations are marked by ‘+’, negative correlations are marked by a ‘-’ sign, and connections which incorporate both positive and negative dimensions are marked by a ‘+/-’ sign.

In the current study, we examined how all these potential factors—level of stress and anxiety, coping strategies, self-efficacy beliefs, and gaps between the needed and received support contribute to teachers’ professional burnout and commitment to work during the 1^st^ and 2^nd^ waves of the pandemic among elementary school teachers. While the relationships between factors such as stress, self-efficacy beliefs, and coping strategy are well-established, there is a lack of understanding of how a health and social crisis such as the COVID-19 pandemic may affect them. Based on the literature reviewed, we developed a theoretical model linking these factors together (Fig 1) and applied structural equation modeling to examine its statistical validity. The model examines how all predictors contribute—both directly and indirectly—to teachers’ commitment to work and to their professional burnout. A secondary goal of this research was to focus on the different types of stressors and support-gaps and their relations with the dependent variables- anxiety, self-efficacy beliefs, burnout and commitment.

Gaining a better understanding of the factors contributing to teachers’ professional burnout and commitment to teaching during a time of global crisis may have important implications for preventing stress-related burnout and applying better coping mechanisms during times of crisis. The pandemic itself is a case study for a scenario with a global impact, and as the literature cited above showed, increased burnout among teachers was observed globally. Although most schools have now returned to in-person teaching routines, understanding of the factors contributing to burnout during crisis may help with preparation for future crises.

## Materials and methods

### Participants and procedure

The study was performed in accordance with the ethical standards of the Hebrew University’s institutional committees and with the 1964 Helsinki Declaration and its later amendments or comparable ethical standards. This study was approved by the ethics committee of the Seymour Fox School of Education, the Hebrew University of Jerusalem (approval number 2021C06). All participants gave written informed consent before participating in any study-related activities.

We conducted a cross-sectional retrospective quantitative research, designed to determine the path to professional burnout and commitment to work among elementary school teachers during the 1^st^ and 2^nd^ waves of the COVID-19 pandemic. Using this design, we could collect data from a relatively large pool of participants at a single point in time.

Data collection took place between January 25th and February 20th, 2021, during Israel’s 3^rd^ wave of the COVID-19 pandemic. A total of 344 teachers were recruited using a “snowball” sampling method, a convenience sampling technique [35], via mailing lists, groups of teachers in digital and social media, and teachers with whom we had prior acquaintance. Using the “snowball” sampling technique, we were able to reach teachers’ populations that are difficult to sample when using other sampling methods.

Sample size calculations were conducted using the G-Power software, based on an expected effect size of Cohen’s d = 0.2, as was found in a recent study [30], for the correlations between the coping approach and the mental state. A sample size of at least 262 participants is required to obtain a power of 0.95 and a significance level of 0.05. Since the dropout rate tends to be relatively high in online studies, we collected data from more participants.

Teachers who met the following inclusion criteria and expressed interest were included in the study: [a] teachers working in elementary, state, or state-religious schools in the Jewish sector. [b] native Hebrew speakers. The exclusion criteria were being on sick leave for more than two weeks or not teaching during this period for reasons other than COVID-19 infection for more than two weeks. We attempted to include a diverse sample in terms of socio-demographic status by advertising in different geographic areas across Israel.

After providing informed consent, participants were given a link to a mobile application and were asked to complete a battery of online questionnaires. The overall completion time for the entire battery was ~25 min. Participants were not directly compensated for their participation in the study. However, participants were asked to provide their email addresses if they wanted to participate in a raffle to win a laptop (four were given to participants).

### Measures

Participants were asked to provide their responses to all questionnaires in relation to the 1^st^ (February through May 2020) and 2^nd^ (June through October 2020) waves of the COVID-19 pandemic in Israel. We measured stressors, needs and sources of support, anxiety, resilience, coping strategy, and self-efficacy beliefs as independent variables, and burnout and commitment to teaching as the dependent variables. Below we provide the full list of measures used.

Since all data collection took place online, we applied the following procedures to maintain the trustworthiness of the data: first, a Google reCAPTCHA was integrated in the application, such that participants were required to click the "I am not a robot" phrase before filling out the questionnaire. This is an acceptable procedure designed to prevent robots from filling out the survey [36]. Next, three easy random mathematical questions were interleaved among the questionnaires (e.g., “2+2”). This was done to make sure that the participants are attentive to the questionnaires and are not providing random answers. Finally, teachers who wanted to participate in the raffle were required to provide their email address, and we verified that the email address given was valid.

Of note, data collected during this study was saved on the secure database only if the participant clicked on "I am not a robot" and approved to continue, completed all questionnaires, and answered the three mathematical questions correctly, and provided a valid email address (in case an email address was provided).

#### Stressors resulting from the COVID-19 pandemic

W used a 15-item questionnaire which is based on Main et al.’s [37] original questionnaire to measure stressors resulting from the SARS pandemic, and was adjusted by Khouri et al. [19] for the COVID-19 pandemic. All items are scored on a 5-level rating scale, ranging from 1 (not at all) to 5 (very much). An exploratory factor analysis with Varimax rotation yielded the following four distinct categories and accounted for 68.5% of the variance: (a) physical concerns, (b) mental health concerns (self and relatives), (c) economic and employment concerns (own, relatives), and (d) concerns related to remote teaching. A total score was derived from the average of all 15 items, as well as sub-scores for each category, with higher scores indicating higher level of concern. The scale had strong internal consistency in our sample (Cronbach’s α = .89).

#### Needs and sources of support during the pandemic

We used a novel questionnaire that was developed specifically for this study. This 32-item questionnaire included questions from two main types: (a) needs—or sources of support that the teachers needed (16 items), and (b) sources of support received by teachers (16 items). Answers to each item were given on a 6-point Likert scale, from 1 (not at all) to 6 (very much). Final scores were derived by calculating the average difference between items in group “a” (needs) and group “b” (sources), as well as separately for each category. Higher scores indicate a higher level of needs or receiving higher assistance compared to what was received. An exploratory factor analysis with Varimax rotation yielded four categories of needs and sources of support, accounting for 60.9% of the variance: (a) the school and the Ministry of Education, (b) remote teaching infrastructure, (c) emotional needs and support, and (d) family and friends. The internal consistency of this scale in our sample was good (Cronbach’s α of .87 and .78 for parts a and b, respectively).

#### State anxiety

State anxiety was assessed using the 20 items assessing state anxiety from the State-Trait Anxiety Inventory (STAI) [38]. Each item is scored on a 4-point scale, ranging from 1 (almost never) to 4 (almost always). Total scores range from 20 to 80 points, with higher scores indicating higher levels of anxiety. The scale also yields a categorical distinction between low (scores between 20–37), moderate (scores between 38–44) and high (above 45) levels of anxiety [39]. The internal consistency of this scale in our sample was high (Cronbach’s α of .92).

#### Psychological resilience

Psychological resilience was measured using the 10-item version of the Connor-Davidson Resilience Scale (CD-RISC) [40]. This scale measures the feeling of resilience and one’s ability to cope with stress. Responses are provided on a 5-point scale ranging from 0 (almost never) to 4 (almost always). Total resilience scores range from 0 to 40 points, with higher scores indicating higher self-reported resilience. The internal consistency of this scale in our sample was adequate (Cronbach’s α = .82).

#### Coping strategies

Coping strategies were measured using the Coping Orientation to Problems Experienced inventory (Brief-COPE) [29]. This 28-item questionnaire measures two categories of coping strategies (see similar use in MacIntyre’s et al. study [29]): 14 items represent ‘approach’ coping strategies and 14 measure ‘avoidant’ coping strategies. Items are rated on a 4-level rating scale, ranging from 0 (not at all) to 3 (very much). The total score in each group is the average of the items, with higher scores indicating higher levels of the coping strategy. The internal consistency in our sample was good (Cronbach’s α = .78 for both avoidant and approach strategies).

#### Teacher’s self-efficacy beliefs

Self-efficacy was measured using the short version (12-item) of the Teacher’s Sense of Efficacy Scale (TSES) [39]. On this scale, teachers were asked to evaluate their likely success regarding remote teaching. We used the overall score (12 to 60 points) based on its high reliability in previous studies (Cronbach’s α = .90) [41] and in the current study (α = .92). Each item rated on a 5-point scale, ranging from 1 (not at all) to 5 (very much) when higher scores indicating higher levels of self-efficacy beliefs.

#### Commitment to teaching

Commitment to the teaching profession was measured using the 9-item Teacher Commitment Scale (TCS) [40]. Items were rated on a 6-point scale, ranging from 1 (not at all) to 6 (very much). Total scores range from 9 to 54 points, with higher scores indicating a higher level of commitment to the teaching profession. The internal consistency of the scale was found to be good in previous studies (Cronbach’s α = .71–.89) [42, 43], as well as in our sample (Cronbach’s α = .84).

#### Professional burnout

Teachers’ burnout from their profession was measured using the Maslach Burnout Inventory (MBI) [1]. Items are rated on a 5-point Likert scale, ranging from 1 (not at all) to 5 (very much). Here, we used the 14 items which measure two components of burnout concerning teacher-student interactions: emotional exhaustion (six items), and personal fulfillment (eight items). The internal consistency of this scale in our sample was good (Cronbach’s α of .76 and .86 for emotional exhaustion and personal fulfillment, respectively).

### Data analysis

IBM SPSS [Statistical Package for the Social Sciences] version 27.0 and IBM AMOS Graphics software version 27.0 were used for statistical analyses. First, descriptive statistics were used to derive participants’ characteristics and study variables. All data were checked for normality and for multivariate outliers. We then used Pearson’s and Spearman’s correlation coefficients to examine the correlations between study variables, and FDR correction with Benjamini-Hochberg method [43] was applied to adjust for multiple testing. After reviewing the correlations, we tested the theoretical model with the factors contributing to burnout and commitment to teaching (see Fig 1), using Structural Equation Model (SEM) [44] with maximum likelihood estimation. Model fit was assessed using the following standard goodness-of-fit indices: chi-square, Comparative Fit Index (CFI), Tucker-Lewis Index (TLI), and Root-Mean-Square Error of Approximation (RMSEA) [45]. A non-significant chi-square, CFI and TLI equal to or greater than .95, and RMSEA equal to or less than .06 are indicative of an acceptable fit. The standardized path coefficients were assessed to examine the statistical significance and directions of path estimate that exist between the variables in the model. Lastly, Pearson’s correlations were used again to zoom-in on the different types of stressors and sources of support and their correlation to the outcome variables. For all analyses, p < 0.05 was considered statistically significant.

## Results

### Characterization of the study sample

Table 1 lists the demographic characteristics of the study sample. A total of 344 elementary school teachers, from 133 different regions and provinces in Israel, participated in this study. There were no missing data points in the study sample. In total, 320 of the 344 participants were female (93%) and 24 males (7%). The age range of participants was 21–69 years (Mage: 40.69 years; SD: 10.85). Most participants were married or in a relationship (82.5%). More than 50% had at least 10 years seniority as teachers.

**Table 1:** Descriptive statistics of the demographic variables in the study sample.

		N = 344	%
**Gender**	Female	320	93.0
	Male	24	7.0
**Age group**	21–35	128	37.2
	36–50	144	41.9
	51–69	69	20.1
**Family status**	Single	34	9.9
	Married/In a relationship	284	82.5
	Divorced	25	7.3
	Widowed	2	0.3
**Professional seniority [years]**	0–5	82	23.8
	5–10	75	21.8
	10–15	53	15.4
	15+	134	39.0

### Descriptive statistics and correlations between measures

Descriptive statistics of the study variables are shown in Table 2. Overall, the mean level of anxiety in the sample was 42.03 ± 11.42, on a scale from 20 (low anxiety) to 80 (high anxiety). More than 60% of participants reported moderate to high levels of anxiety (total score of 38–80) during the pandemic: 21.51% experienced moderate levels of anxiety (total score of 38–44; M = 41.35 ± 11.41) while 39.83% of them experienced high anxiety (total score of 45 and over; M = 53.29 ± 7.46). The overall mean level of psychological resilience in the study sample was M = 29.7 ± 7.0 on a scale from 0 (low resilience) to 40 (high resilience).

**Table 2:** Descriptive statistics and Pearson correlational analyses^a^ of the relationship between study variables.

	N = 344	Mean (SD)	1, r	2, r	3, r	4, r	5, r	6, r	7, r	8, r	9, r
**Coping**	**1. Avoidant-coping**	13.20 (6.64)	1								
	**2. Approach coping**	24.01 (5.95)	.32***	1							
	**3. Gap in Support**	0.52 (1.07)	.13*	.05	1						
	**4. Stressors**	2.91 (0.84)	.44***	.15**	.36***	1					
	**5. Psychological Resilience**	29.73 (7.01)	-.26***	.24***	-.06	-.19***	1				
	**6. Self-efficacy**	5.06 (1.22)	-.05	.21***	-.14*	-.03	.33***	1			
	**7. State anxiety**	42.03 (11.43)	.55***	-.02	.32**	.57***	-.43***	-.24***	1		
**Burnout**	**8. Emotional exhaustion**	20.79 (6.82)	.37***	.07	.36***	.41***	-.25***	-.20***	.58***	1	
	**9. Personal fulfillment**	20.14 (5.36)	-.10	.11	-.29***	-.24***	.25***	.28***	-.34***	-.54***	1
	**10. Commitment**	3.87 (1.00)	-.13*	.07	-.39***	-.22***	.24***	.30***	-.38***	-.56***	.77***

^a^ All reported p-values were adjusted using FDR correction;

* *p* < .05;

** *p* < .01;

*** *p* < .001.

As a first step towards forming the model, we first calculated the correlations between predictor variables and between predictors and outcomes (see Table 2). As expected, both commitment to teaching and personal fulfillment (the 1^st^ factor of burnout) were positively correlated with the predictors of psychological resilience and self-efficacy beliefs, and negatively correlated with state anxiety. Furthermore, commitment to teaching and personal fulfillment had a significant negative correlation with the gap in support (i.e., the gap between the support needed and the support received) and with stressors. In other words, the larger the gap between needs and provided support, and the higher the level of stressors, the lower the commitment to teaching and the sense of fulfillment. In addition, commitment to teaching had a significant weak negative correlation with avoidant-coping, such that more use of avoidant coping was associated with less commitment. No such correlation was found with the ‘approach’ coping style.

The 2^nd^ factor of burnout—emotional exhaustion—was negatively correlated with resilience and with self-efficacy beliefs, such that lower levels of psychological resilience and perceived self-efficacy beliefs were associated with higher levels of emotional exhaustion. As expected, emotional exhaustion was positively correlated with avoidant-coping, gap in support, and with all stressors, such that higher levels of emotional exhaustion were associated with higher levels of avoidant-coping, reduced support-gap, and with higher levels of external stressors.

We further examined the correlation between professional seniority (i.e., the number of years as a teacher) and all other variables using Spearman’s correlations. Professional seniority was weakly negatively correlated with anxiety, with emotional exhaustion, and with the gap between needed and provided support (r_s_(344) = -.13; -.12; -.11, respectively; all p values < .05), and weakly positively correlated with commitment to teaching and with personal fulfillment (*r*_s_(344) = .11; .15, respectively, all *p* values < .05).

### Validation of the theoretical model using SEM

**Fig 2:**
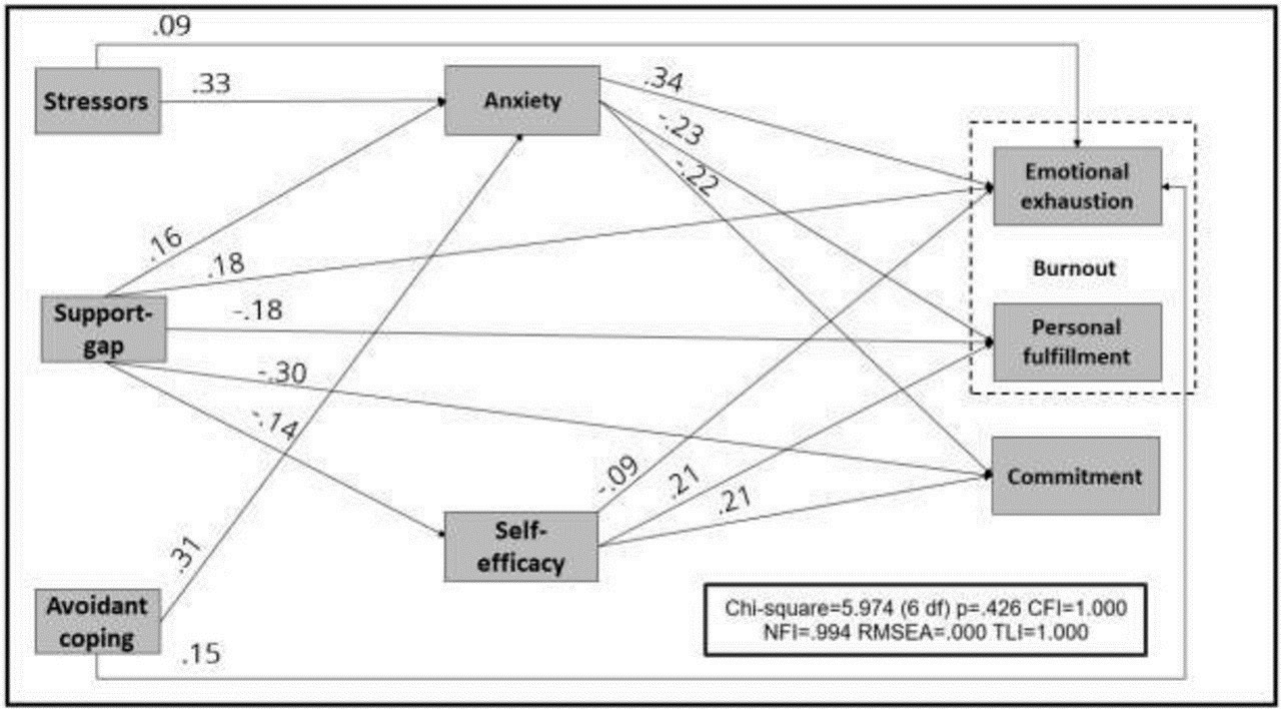
A structural equation model (SEM) analysis of the effect of avoidant-coping, support-gap, stressors (total), self-efficacy, and state anxiety on professional burnout (comprised of emotional exhaustion and personal fulfillment) and on commitment to teaching.

Our first goal was to examine the direct effects of model predictors—namely, stressors, coping strategies, seniority, resilience, and support—on professional burnout and commitment to teaching. In addition, studied their indirect effects through anxiety and self-efficacy beliefs. To further examine the theoretical model, we used Structural Equation Modeling (SEM). We excluded the variables with no significant direct or indirect effect on the dependent variables: professional seniority, resilience, and approach coping. The final model is given in Fig 2. All fit indices indicate a suitable fit of the model to the data (Χ^2^(6) = 5.974, p = .426, CFI = 1.00, NFI = .99, RMSEA = .00, and TLI = 1.00).

The gap in support had a direct and significant effect on *all* other variables. Specifically, insufficient levels of support directly affected burnout (personal fulfillment and emotional exhaustion) and commitment to teaching. Support-gap also indirectly affected burnout and commitment to teaching, via its effect on self-efficacy beliefs and on anxiety. In other words, insufficient support (less received than desired) directly led to lower levels of professional commitment and to higher levels of burnout, and indirectly, by contributing to the reduction in self-efficacy beliefs and higher levels of anxiety.

Both stressors which were associated with the COVID-19 pandemic (i.e., health concerns, dealing with remote teaching) and using the avoidant-coping style had similar and small direct effect on the emotional exhaustion component of burnout, but not on personal fulfillment nor on commitment to work. Interestingly, both variables—stressors and avoidant-coping—had strong indirect effects on burnout and professional commitment, via their strong positive effects on anxiety (0.33 and 0.31 for stressors and avoidant-coping, respectively).

Finally, both anxiety and self-efficacy beliefs significantly contributed to the two components of professional burnout and to commitment to teaching. Specifically, higher levels of anxiety reduced personal fulfillment and commitment to teaching, and increased emotional exhaustion, while higher levels of self-efficacy beliefs had the opposite effect.

### COVID-19 related stressors in relation to outcome measures

A secondary goal of the study was to better understand different factors related to the pandemic in relation to well-being and burnout among teachers. More specifically, we aimed to focus on different sources of stress and the gap in support, and their relations with anxiety, self-efficacy beliefs, professional burnout and commitment. For this, we calculated the correlations between all types of stress and support-gaps with all outcomes (see Table 3).

**Table 3:** Descriptive statistics and Pearson correlational analyses^a^ of the relationships between different types of stressors and sources of support-gap resulting from the COVID-19 pandemic and other variables.

					Burnout		
		Mean (SD)	Self-efficacy	Anxiety	Emotional Exhaustion	Personal fulfillment	Commitment
**Stressors**	**Physical health**	3.07 (1.04)	.06	.24***	.16**	-.09	-.08
	**Mental health**	2.74 (1.35)	-.04	.57***	.36***	-.23***	-.17**
	**Economic and employment**	3.02 (1.19)	.09	.29***	.24***	-.10	-.09
	**Remote teaching**	2.82 (1.09)	-.19***	.49***	.41***	-.26***	-.29***
**Support-gap**	**Family and friends**	0.24 (1.44)	-.10	.27***	.26***	-.17***	-.24***
	**Emotional**	0.84 (1.22)	.01	.18***	.17***	-.15**	-.20***
	**Remote teaching**	0.48 (1.74)	-.18***	.25***	.33***	-.28***	-.39***

^a^ All reported p-values were adjusted using FDR correction;

** *p* < .01;

*** *p* < .001.

We found that stressors related to remote teaching and the gap in support of remote teaching were significantly correlated with all other outcome measures. Higher levels of stress from remote teaching were associated with increased anxiety and with emotional exhaustion. In addition, a larger gap between the support needed and received for remote teaching was associated with lower levels of commitment to teaching. In general, all stressors and support-gaps were positively correlated with anxiety and with emotional exhaustion.

## Discussion

In this study, we examined the factors which contributed to teachers’ burnout and commitment to teaching during the COVID-19 pandemic. For this, we collected information from 344 elementary school teachers, assessing their mental health, concerns, and resources during the pandemic. Using SEM analysis, we found that the stressors, gaps in support, and coping strategies all contributed to teachers’ burnout, both directly and indirectly, via their effect on anxiety and self-efficacy beliefs. The gaps in support further affected teachers’ commitment to work. A closer look at the different stressors and sources of support and their relations with other variables revealed that the most significant predictors of professional burnout and commitment were stressors and gaps in support which were specifically related to remote teaching. Another contributor was the use of avoidant-coping strategies, which was associated with increased anxiety and burnout and decreased self-efficacy beliefs and commitment.

### The contribution of support-gaps, stressors, and coping strategy

A main finding in our study is the fact that insufficient support (i.e., a larger gap between needed and received support) contributed both directly to lower levels of professional commitment and to higher levels of burnout, as well as indirectly, by affecting both self-efficacy beliefs and anxiety. This novel finding is generally in line with previous literature, showing that a supportive environment increases the likelihood of teachers remaining in their job for extended periods of time [46]. Specifically, teachers rated the support they received from the school’s principal as a critical factor contributing to a feeling of professional satisfaction [47]. Our study demonstrated the importance of support from schools during emergency times such as the pandemic, even when teaching is done from home. Our results further showed that among the different sources of stress and support, the support from schools is even more critical than support from family and friends. This finding suggests that stakeholders and schools should focus on supplying support to teachers on normal days and particularly during crisis times.

In addition to support, we further found that stressors associated with the pandemic (e.g., health concerns, remote teaching) as well as avoidant-coping styles had direct effects on emotional exhaustion (one of the two components of burnout), and indirect effects on burnout and on professional commitment, via their effects on anxiety. Increased stress during the pandemic has been shown in multiple studies to date, and a recent meta-analysis concluded that 30% of teachers experienced high levels of stress during the pandemic [48]. In addition, a recent study conducted among Israeli teachers found that more than half of them experienced high levels of stress, which were associated with increased burnout and a desire to leave the profession [14]. Here we further found that teachers’ burnout was in addition affected by their use of a maladaptive coping strategy, sources of support, and their self-efficacy beliefs, suggesting that tools to strengthen these supports should be provided to teachers by schools ahead of time. In addition, our zooming in on different stressors revealed that stress relating to remote teaching was the most significant one of all the stressors, suggesting that the challenges of teaching were even more salient than the direct effects of the pandemic.

The use of a particular coping strategy to handle stressful situations also contributed to burnout. Specifically, we found that the use of an avoidant-coping strategy was associated with higher levels of stress and anxiety and was associated with increased burnout, and with a reduced commitment to teaching. This finding is consistent with a recent study by MacIntyre and colleagues, in which the authors reported that an avoidant-coping strategy was associated with increased negative emotions of anger, sadness, and loneliness in teachers during the pandemic [29]. Here, we further show that coping strategies are not only related to changes in mental health but also contribute to burnout and commitment to work. Interestingly, however, the more adaptive coping strategy (approach-coping) was not associated with burnout or with commitment in our study. The fact that the study by MacIntyre and colleagues did find effects of this strategy on positive emotions may indicate that while this type of strategy is beneficial for positive emotions in personal life, it may not have a significant effect on work-related outcomes. It may be that professional burnout and commitment to work are less related to a positive attitude toward the pandemic, such as the approach coping strategy. More research is needed to better understand the potential contribution of this type of strategy to burnout among teachers.

### Anxiety and self-efficacy beliefs as contributors to burnout

More than 60% of the teachers in our sample reported medium to high levels of anxiety during the pandemic. This finding is aligned with those of recently-conducted studies [e.g., 13, 27, 48] and of recent meta-analyses showing high levels of anxiety during the pandemic both in the general population and specifically among teachers [49, 50]. Here, we further show that higher levels of anxiety contributed to a reduction in personal fulfillment and commitment to teaching and increased emotional exhaustion among teachers, indicating that teacher mental state had a significant impact on their professional functioning. The indirect relations between support, anxiety, burnout, and commitment to teaching in our model, suggest that sufficient support from school could alleviate anxiety which in turn would have led to less burnout and better commitment to teaching during the pandemic.

Gaps in support also influenced teacher self-efficacy beliefs, which in turn have led to increased burnout and decreased commitment. Several studies demonstrated that during pandemic times, teacher self-efficacy beliefs were reduced compared with pre-pandemic times [e.g., 27]. This reduction in self-efficacy beliefs appears to be related to remote teaching. Teachers who were only teaching virtually reported the lowest levels of self-efficacy beliefs, as compared to teachers who were teaching in a hybrid or all-in-person form [27]. Potentially, the requirements associated with remote teaching, including learning new technologies and adapting lesson plans for virtual and hybrid instruction, have a significant effect on self-efficacy beliefs. In another study, self-efficacy beliefs were also found to mediate the association between difficulties stemming from remote teaching and perceived stress [51]. Collectively, these findings and our results demonstrate the difficulties in adopting new forms of teaching. They may also suggest that support from the school in adopting new methods of teaching during the pandemic could contribute to teacher self-efficacy beliefs. A strong sense of self-efficacy leads to less burnout and a greater commitment to teaching. Strategies for increasing self-efficacy beliefs among teachers could therefore be employed to reduce burnout and increase commitment, especially during crisis times [52].

### Psychological resilience

Psychological resilience entails better recovery from adversity and a better ability to regulate negative emotions [53]. Indeed, individuals with high resilience adapt better to stressful situations [54]. Our findings show that higher levels of resilience alleviated teachers’ stress due to remote teaching. We claim here that teachers who have high levels of self-reported resilience can better adopt new teaching methods and frequent changes between methods. This finding is also in line with previous findings which pointed to the relationship between resilience, professional functioning, self-efficacy beliefs, burnout, and stress among teachers, during non-pandemic times [55]. Interestingly, however, the effect of resilience in our model was weaker, secondary to more prominent variables such as support-gap and stressors. It may be that during emergency times the effect of internal factors such as resilience are weaker than the effect of external factors such as support. Future studies should therefore address the question of psychological resilience and examine its relationship with teachers’ burnout during times of distress.

### Source of stress and support during the pandemic

To examine the factors associated with teachers’ stress during the pandemic, we assessed four different categories of stressors–- stressors related to remote teaching, health worries, financial concerns, and occupational worries. Our results show that all these categories were significant sources of stress for teachers during the pandemic, all directly affecting their professional burnout. The effect of stressors on burnout and their commitment to teaching was also expressed indirectly, via its effect on anxiety and self-efficacy beliefs. This finding is consistent with the literature, generally showing that stress contributes to professional burnout. Specifically, previous studies found that even during non-pandemic times, teachers deal with many professional stressors leading to stress at work and in their personal life, which in turn lead to professional burnout [56].

Among the four stressors examined, our findings indicate that stress related to remote teaching was the most significant one. Remote teaching that was enforced during the long lockdown periods included new challenges related to online teachings, such as the use of novel technology, teaching from home while having young children, sharing computers between family members, and the like. Our results are consistent with those of recent studies, showing that one of the main stressors that led to burnout among teachers during the pandemic was new teaching demands due to the transition to online teaching [32]. While we hypothesized that teachers’ seniority will affect this factor, no such effect was found in our study. It may be that remote teaching was novel enough to both new and senior teachers alike, hence no effect of seniority was found. In addition to the difficulties brought about by the switch to online teaching, another study found that teachers believed that the online platform prevented teachers from teaching the regular curriculum [47]. Furthermore, a recent study found that even teachers with relevant technological skills reported decreased well-being [57]. Our results further suggest that online teaching constituted the primary stressor among teachers, even compared to health-related stressors, leading to more significant burnout and reduced commitment to teaching.

A novel finding from our research is that insufficient support from schools increased teacher burnout and decreased their commitment to work. We examined the gaps between the support that teachers felt they needed, compared to the support they received, and found that the highest gap was in the support related to remote teaching. This gap significantly contributed to lower levels of commitment and self-efficacy beliefs and higher levels of anxiety and professional burnout. A previous study found that altogether, the use of new information and communication technologies, work/family conflict, social support, and workload related to distance education, have led to increased burnout [12]. Our study further shows that remote teaching did not constitute merely a technical challenge or exhaustion from an increased workload, but instead, has led to stress and a specific need for support from the school. Specifically, more significant support from the school regarding remote teaching might have reduced adverse feelings among teachers such as anxiety. Interestingly, a recent study reported that not just the pandemic—but also returning to teaching in the classroom after it—was accompanied by high stress and anxiety among teachers [58]. This finding may suggest that stressors related to remote teaching may also stem from frequent changes and a lack of stability and consistency. One conclusion is that support from the school should supply teachers not only with technical skills but also emotional skills to deal with this kind of situation.

### Broader implications

This study is rooted in specific educational, technological, social, and cultural circumstances in Israel which affect Israeli schools and remote teaching. Nevertheless, the literature outlined above along with our findings demonstrates a universal effect of COVID-19 on teachers around the globe. Despite the different cultures and teaching methods, the global transition to remote teaching universally led to a reduced commitment to teaching, and increased stress, anxiety, and burnout for teachers. The results of the current study therefore further contribute to this body of knowledge, specifically highlighting the need for better preparation and training for teachers in novel remote teaching methods.

This study has important implications in two dimensions—technical and psychological. At the technical level, stakeholders should make efforts to strengthen remote teaching skills among teachers and ensure that teachers have all the necessary facilities for remote teaching and to support a smooth transition to remote teaching when this is required [59]. In addition, help can be provided in the form of a strategy of reframing, which aims at leading to an approach coping, instead of the use of avoidant coping. The contribution of mental health-related factors—such as anxiety and distress—to burnout and commitment, calls for providing better and stable support for teacher populations, especially during crisis times. There is a need to provide teachers with a place to express their feelings at such times, exposing administrators and stakeholders to teachers’ actual needs [60].

### Study limitations

Our study has several limitations which may affect the generalizability of the findings. First, data collection was done retrospectively, i.e., participants were asked to address their feelings about the 1^st^ and 2^nd^ waves of the pandemic during the 3^rd^ wave. Such retrospective self-reports may be biased in multiple ways and are limited to a subjective point of view. Importantly, during the 3^rd^ wave of the pandemic, unlike the uncertainty of the first two waves, people may have felt more depression or despair, which is more characteristic of a chronic, rather than an acute state [61].

In addition, the design of this study was cross-sectional, and as such, no causal or sustained effects could not be addressed. This cross-sectional design further limited our ability to assess the contribution of effects not related to COVID-19 on professional burnout among the teachers in our sample. Second, our study sample included elementary school teachers, which limits their generalizability to middle and high school teachers. Although we aimed for a diverse sample, the majority of the sample included women, which is also similar to their proportion in the teaching profession. Given that past research indicated that teachers’ mental health is significantly related to gender [62], the results may be biased in this respect. Future research with long-term follow-up and a diverse sample of teachers from different educational systems and genders should be conducted to strengthen and validate the current findings.

Finally, we should note that the study only examined a limited set of potential contributors to burnout and professional commitment, considering the feasibility of the remote application of the study. Other factors which may have affected burnout such as socioeconomic status were not considered here, and should be examined in future studies.

## Conclusions

We found that gaps between the needed and received support had a direct effect on teacher burnout and commitment, and an indirect effect through anxiety and self-efficacy beliefs. Stress relating to remote teaching and support-gaps regarding remote teaching were the most significant of all the stressors and sources of support. These findings demonstrate the significance of remote teaching as the main cause of stress and professional burnout and suggest that proper preparation of teachers and support by schools can have a significant effect on teachers’ mental and professional well-being.
